# The combined signatures of programmed cell death and immune landscape provide a prognostic and therapeutic biomarker in the hepatocellular carcinoma

**DOI:** 10.3389/fchem.2024.1484310

**Published:** 2024-11-12

**Authors:** Wanghu Liu, Yan Huang, Yang Xu, Xuanji Gao, Yifan Zhao, Simin Fan, Yuanzhi Geng, Shajun Zhu

**Affiliations:** ^1^ Department of General Surgery, Affiliated Hospital of Nantong University, Medicine School of Nantong University, Nantong, China; ^2^ Department of Hepatobiliary and Pancreatic Surgery, Affiliated Hospital of Nantong University, Medical School of Nantong University, Nantong, China; ^3^ Department of Nursing, Affiliated Hospital of Nantong University, Nantong, China; ^4^ Medicine School of Nantong University, Nantong, China

**Keywords:** tumor microenvironment, hepatocellular carcinoma, programmed cell death, immunotherapy, prognosis

## Abstract

Hepatocellular carcinoma (HCC) ranks as the fourth most common cause of mortality globally among all cancer types. Programmed cell death (PCD) is a crucial biological mechanism governing cancer progression, tumor expansion, and metastatic dissemination. Furthermore, the tumor microenvironment (TME) is critical in influencing overall survival (OS) and immune responses to immunotherapeutic interventions. From a multi-omics perspective, the combination of PCD and TME could help to predict the survival of HCC patient survival and immunotherapy response. Our study analyzed variations in the PCD- and TME-classifier used in the classification of HCC patients into two subgroups: PCD high-TME low and PCD low-TME high. In the following step, we compared the tumor somatic mutation (TMB), immunotherapy response, and functional annotation of both groups of patients. Lastly, Western Blot (WB) were conducted. The immunohistochemistry (IHC) was performed on the Human Protein Atlas (HPA). In the PCD–TME classifier, 23 PCD-related genes and three immune cell types were identified. Patients’ prognoses and responses to therapy could be accurately predicted using this model. The findings of this study provide a new instrument for the clinical management of HCC patients, and they contribute to the development of accurate treatment strategies for these patients.

## 1 Introduction

HCC is responsible for more than 85% of liver malignancies and ranks as the fourth most common cause of cancer-related mortality worldwide (Villanueva, 2019). In recent decades, there has been a significant rise in the global incidence of HCC (Yang et al., 2019). Following primary hepatic resection, patients diagnosed with HCC in China have a median survival of 47 months and a 5-year survival rate of 45%. However, recurrence occurs in 54% of cases, leading to a 24% decrease in 5-year survival and a reduction of 54 months in median survival (Tabrizian et al., 2015). Although cancer immunotherapy has significantly advanced cancer treatment, it is effective for only a small subset of patients (Pinter et al., 2021). Consequently, there is a significant focus on the identification of novel biomarkers for prognosis and therapeutics. Nowadays, the prognosis for HCC is on the basis of clinical classification (Satala et al., 2021) and staging systems (Llovet et al., 1999), which consider factors such as lymph node involvement, metastasis, and liver function. Because of the high heterogeneity of tumors (Hung and Wang, 2019), patients with similar clinical characteristics often experience different outcomes. It is essential to note that HCC is a heterogeneous tumor characterized by a diverse array of oncogenic pathways (Llovet et al., 2018). Therefore, it is imperative to conduct additional thorough research and discover novel biomarkers to forecast the prognosis and therapeutic of HCC patients effectively.

PCD plays a critical role in the development of organisms. Various researchers have found Pyroptosis, Apoptosis, Autophagy, Ferroptosis, Cuproptosis, Necroptosis, Alkaliptosis, Oxeiptosis, NETosis, Parthanatos, Entotic, and Lysosome-dependent cell death are classical pathways of cell death (Tang et al., 2019). Pyroptosis is triggered by the activation of inflammatory caspases that bind to Gasdermin proteins, forming pores in the plasma membrane and leading to cell death (Kayagaki et al., 2015; Shi et al., 2015). In HCC, pyroptosis has shown potential in enhancing anti-tumor immune responses, and GSDME-mediated pyroptosis is being explored as a novel therapeutic approach (Zhang et al., 2020). In contrast, apoptosis is a non-inflammatory PCD pathway characterized by caspase activation, resulting in cell shrinkage, nuclear fragmentation, and DNA degradation (Wyllie, 1987). Dysregulation of this process is a hallmark of cancer cells (Hanahan and Weinberg, 2011). Cells often evade apoptosis by overexpressing anti-apoptotic proteins like BCL-2. Targeting this pathway with BCL-2 inhibitors is being explored to restore apoptotic sensitivity in HCC (Carneiro and El-Deiry, 2020). Autophagy involves the degradation of cellular components through the formation of autophagosomes, which merge with lysosomes (Kroemer and Levine, 2008; Tasdemir et al., 2008). Ferroptosis is an iron-dependent form of PCD that disrupts redox homeostasis (Ursini and Maiorino, 2020). It is marked by mitochondrial membrane damage and iron accumulation (Tang et al., 2021). Ferroptosis is being studied as a therapeutic target, particularly in overcoming therapy resistance related to oxidative stress and iron metabolism. Cuproptosis, caused by copper ions interacting with thioketone proteins, leads to protein aggregation and cell death (Tsvetkov et al., 2022). Necroptosis, distinct from conventional apoptosis, is a pro-inflammatory form of cell death mediated by RIPK1 and RIPK3, leading to MLKL-mediated membrane rupture (Tanzer et al., 2017). Necroptosis is being investigated as a strategy to enhance immune responses and combat tumor progression. As research on programmed cell death continues to evolve, these pathways present promising therapeutic targets for hepatocellular carcinoma. By leveraging strategies to induce Pyroptosis, Ferroptosis, and restore Apoptosis, and by further exploring the roles of Cuproptosis and Necroptosis, innovative treatments may be developed to improve patient outcomes and combat therapy resistance in HCC.

Over an extended period, PCD has been proven to be integral to malignant tumor progression and metastasis. The development of malignant tumor cells necessitates evading different types of cell death mechanisms (Su et al., 2015; Chen et al., 2023). Nevertheless, there remains a deficiency in the comprehensive understanding of the correlation between PCD and HCC. Thus, it is imperative to utilize array-based databases to pinpoint genes linked to survival in order to forecast prognosis and inform individualized treatment strategies (Wang et al., 2023). The purpose of our research is to systematically construct a PCD-tumor microenvironment (TME) classifier that combines PCD and TME for the aim of forecasting prognosis and immunotherapy response. Our findings in this study suggest that the integration of a PCD-TME classifier has the potential to improve understanding of tumor-specific biology, leading to significant implications for personalized treatment strategies in clinical practice.

## 2 Materials and methods

### 2.1 Data sources and analysis platforms

The TCGA database (https://portal.gdc.cancer.gov) was utilized to procure RNA sequencing and clinical information for 374 HCC samples. Additionally, five single-cell RNA sequencing (scRNA) data of HCC were downloaded from GSE242889 (Li et al., 2024) (https://www.ncbi.nlm.nih.gov/geo/query/acc.cgi) to visualize PCD scores within immune cells. External validation data was sourced from ICGC-LIRI-JP (https://dcc.icgc.org/projects/LIRI-JP) and GSE10143 (Hoshida et al., 2008). Three analytical platforms were employed in the comprehensive analysis. The Metascape online tool (https://metascape.org/) was utilized for the analysis of functional annotations (Zhou et al., 2019), while TIDE (http://tide.dfci.harvard.edu/) was conducted for the prediction of immunotherapy responses (Jiang et al., 2018). Additionally, Proteomaps (https://proteomaps.net/) was performed to illustrate the composition of protein in each groups (Liebermeister et al., 2014). Finally, the Human Protein Atlas (HPA) (https://www.proteinatlas.org/) was consulted for immunohistochemical (IHC) staining evaluation comparing healthy and HCC samples.

### 2.2 Data preprocessing

RNA-seq data normalization was performed utilizing the “DESeq2” R package, while background correction and normalization of microarray data were done by the “affy” package. ScRNA-seq data was normalized by utilizing the “NormalizeData” function within the “Seurat” package.

### 2.3 Quantification of PCDs and TME cells

PCD-associated genes were obtained from existing literature sources (see Supplementary Table 1). Utilizing the CIBERSORT tool, a deconvolution algorithm was employed to calculate 22 different immune cell types using bulk-seq data (Chen et al., 2018). In order to maintain consistency and accuracy in our gene expression data, we adhered to standard preprocessing procedures for normalizing RNA-seq and microarray data. The TME score was determined by calculating enrichment scores generated by CIBERSORT.

### 2.4 Establishment of the PCD score

We conducted univariate Cox regression analysis with bootstrap resampling (1,000 iterations) to identify potential prognostic PCDs related to OS in the TCGA-LIHC dataset. To further refine the selection of prognostic PCDs, we employed the Least Absolute Shrinkage and Selection Operator (LASSO) regression analysis using the “glmnet” R package. A bootstrap algorithm (resampling = 1,000) was employed in a multivariate Cox regression analysis to identify the most correlated PCDs with prognosis. To the stability of the results, we fixed the bootstrap coefficient of each included PCD:

$$bootstrap\;coefficient=\frac{coefficient}{bootstrap\;standard\;deviation}$$

The PCD score was calculated using the formula below:

$$PCD\;SCORE=\sum_{i=1}^{n}bootstrap\;coefficient\;\left(included\;{PCD}_{i}\right)\times expression\;level\;\left(included\;{PCD}_{i}\right)$$

We used the median as a cutoff point for categorizing samples into high- and low-score groups. The TCGA–LIHC cohort investigated survival differences among two PCD score groups by the “survival” package.

### 2.5 Establishment of the TME score

The TME score was determined by quantifying the presence of immune cells in 22 subtypes of HCC utilizing the CIBERSORT algorithm. Subsequent survival analysis was conducted for individual patients based on the immune cell infiltration, Prognostic immune cells have been identified as those demonstrating differential overall survival rates among various subgroups. Additionally, the prognostic immune cells’ bootstrap coefficient was computed through multivariate Cox regression analysis (resampling = 1,000). The TME score was defined as:

$$TME\;SCORE=\sum_{i=1}^{n}bootstrap\;coefficient\;\left(prognostic\;{immune\;cell}_{i}\right)\times infiltration\;level\;\left(prognostic\;{immune\;cell}_{i}\right)$$

Patients were categorized into two subgroups, TME low and TME high, based on their median TME score. This stratification was done in order to conduct a survival analysis and evaluate differences in OS between the two subgroups. Subsequently, the PCD–TME classifier was formulated by integrating the PCD and TME scores. Within the study, patients with HCC were segregated into four distinct subgroups according to their PCD and TME scores: PCD high-TME low, PCD low-TME low, PCD high-TME high, and PCD low-TME high. To simplify the PCD-TME classifier for clinical application, PCD high-TME high and PCD low-TME low were amalgamated into a single category labeled as Mixed due to their less divergence. The study proceeded with a survival analysis to examine variations in OS across the three groups. Following this, the “timeROC” and “survivalROC” R packages were applied to evaluate the efficacy of the PCD-TME classifier by determining the area under the curve (AUC) of receiver operating characteristic (ROC) curves at 1-, 3-, and 5-year time points.

### 2.6 Independence and robustness of the PCD–TME classifier

We performed survival analysis in the TCGA-LIHC cohort to examine variations in OS among subgroups. The PCD-TME classifier was evaluated for its potential as an autonomous prognostic indicator for hepatocellular carcinoma (HCC) within the TCGA-LIHC through Cox regression analyses. Additionally, the findings of this study were corroborated through validation in the ICGC-LIRI-JP.

### 2.7 Enrichment analysis of the PCD–TME classifier

Gene set enrichment analyses (GSEA) were carried out to identify pathways linked to low PCD-high TME and high PCD-low TME. Genes with comparable expression profiles were clustered using weighted gene co-expression network analysis (WGCNA) (Costanzo et al., 2016; Giulietti et al., 2016) through an unsupervised analysis approach. Subsequently, a Metascape analysis was conducted to visually represent the enrichment results for the genes that were identified as key modules by WGCNA.

### 2.8 Analysis of TMB, KEGG pathways, and functional annotations

An effective anti-tumor immune response can be initiated through tumor mutational burden (TMB) (Meléndez et al., 2018), leading to prolonged clinical outcomes. TMB levels were compared among subgroups by calculating individual TMB scores for samples in the TCGA-LIHC cohort using established methodologies. Subsequently, hub genes exhibiting differences among high PCD-low TME and low PCD-high TME were identified and analyzed. TMB scores for each tumor were also computed following established protocols. The differential gene expression analysis was utilized by the R package of “limma.” Furthermore, Proteomaps were constructed utilizing a web-based tool. The R package “clusterProfiler” was employed to conduct the analysis of the Kyoto Encyclopedia of Genes and Genomes (KEGG) pathways across all cohorts (Liebermeister et al., 2014).

### 2.9 Analysis of scRNA-sequencing PCD-TME scores

In the analysis of scRNA sequencing, cells were filtered based on the criteria of detecting between 300 and 5,000 genes, with mitochondrial genes accounting for less than 30% of the total. Data normalization was performed using the LogNormalize method, and the top 2,000 most variable genes were identified for downstream analysis. Dimensionality reduction was conducted using Principal Component Analysis (PCA), and the first 20 components were used to cluster cells. Batch effects were corrected using the Harmony algorithm. The “inferCNV” package was used to identify malignant cells. Additionally, intercellular communication patterns were elucidated using the “CellChat” package.

### 2.10 Western blot analysis

Following the quantification of protein concentration, the protein sample underwent electrophoretic separation on 12% SDS−PAGE gels and subsequent transfer onto 0.45-mm PVDF membranes (Millipore). Subsequently, the membranes were subjected to incubation with HTRA2 Antibody (AF1855, Beyotime) at 4°C overnight, followed by HRP-conjugated secondary antibodies (AS014, ABclonal). The primary antibody was diluted at a ratio of 1:1,000, while the secondary antibody was diluted at a ratio of 1:3,000. Finally, images were captured using a ChemiDoc Imaging System (Bio-Rad, United States), and quantitative analysis was performed using ImageJ. Student’s t-test was used for data comparisons between JHH-7 HCC cells and L-O2 normal liver cells.

### 2.11 Quantitative real-time PCR

Total RNA was extracted from JHH-7 HCC cells and L-O2 normal liver cells using the RNA Isolation Kit (R0017M, Beyotime). RNA quantity and concentration were measured using a NanoDrop 2000 spectrophotometer (Thermo Scientific, United States). Reverse transcription of total RNA to cDNA was performed using the BeyoRT™ III M-MLV Reverse Transcriptase Kit (D7176M, Beyotime). Quantitative real-time PCR (qRT-PCR) was then conducted using the Taq Pro Universal SYBR qPCR Master Mix Kit (Vazyme, China). The cycling threshold (Ct) for HTRA2 was recorded, and the relative expression of HTRA2 mRNA was calculated using the 2^−ΔΔCT^ method, with appropriate controls. The primers used in the qRT-PCR protocol are listed in Supplementary Table 2.

### 2.12 Statistical analysis

Statistical analyses were performed with various methods, including Cox regression analyses, log-rank tests, Wilcoxon rank-sum tests, Student’s t-tests, and Fisher’s exact tests in R 4.1.1. Multiple testing correction was implemented using the Bonferroni method for comparisons involving multiple groups. Statistical significance was set at *p* < 0.05 unless specially indicated. * P < 0.05 ; ** P < 0.01 ; *** P < 0.001 .

## 3 Results

### 3.1 Construction of the PCD score in TCGA-LIHC

**FIGURE 1:**
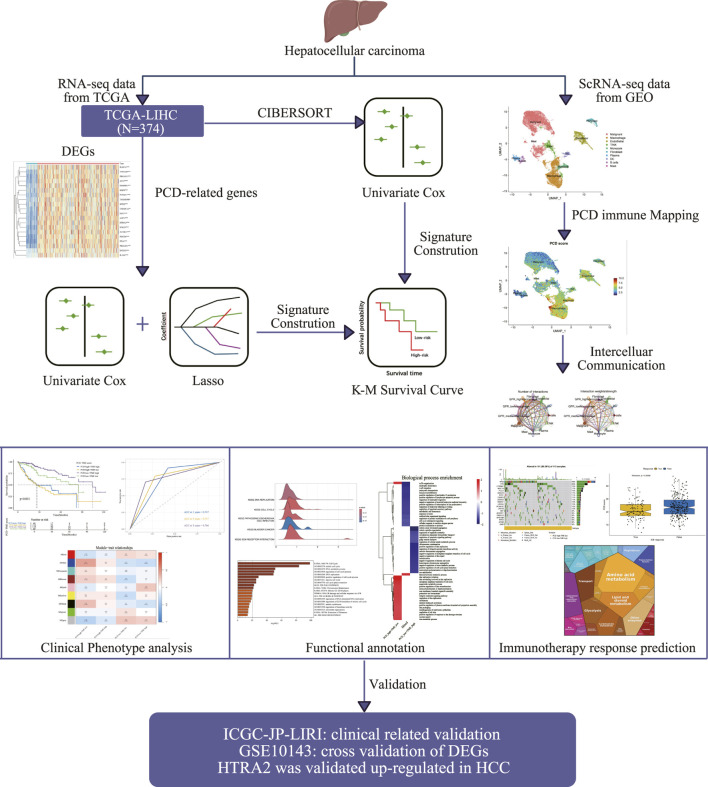
The schematic diagram illustrates the creation and comprehensive assessment of the PCD-TME classifier.

**FIGURE 2:**
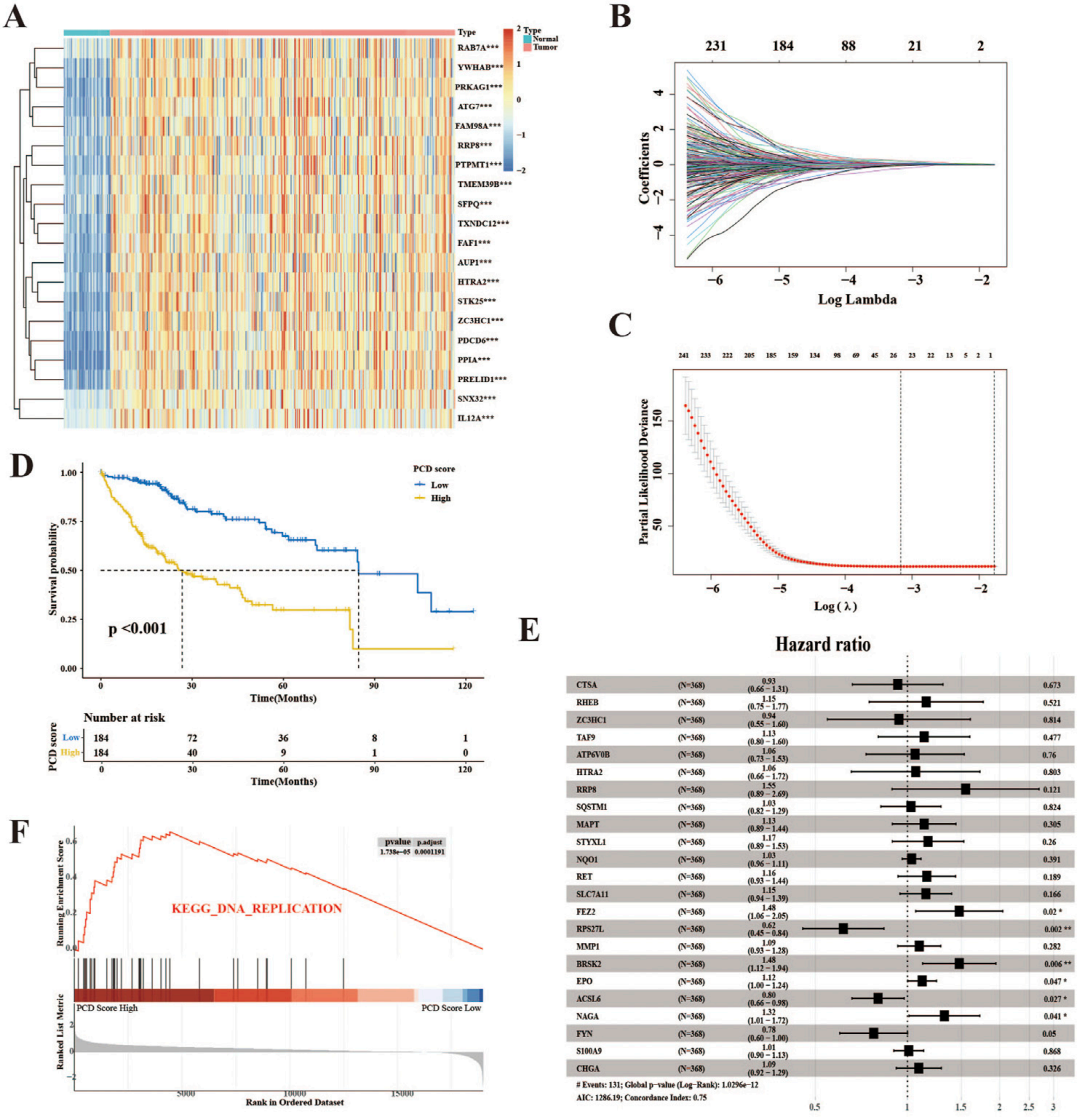
The development of the PCD score in the TCGA-LIHC cohort. **(A)** The heatmap displays the top 20 prognostic genes and DEGs selected for PCD score establishment. **(B)** The determination of the optimal value of λ for the LASSO analysis. **(C)** LASSO regression was used to analyze the coefficient profiling of 23 genes within the TCGA-LIH group. **(D)** K–M survival analysis for HCC patients divided into the low- and high-score groups using PCD scores. **(E)** The forest plot illustrates the results of a multivariate Cox analysis conducted on the genes included in the study. **(F)** The top enriched signaling pathway in the PCD high subgroup according to GSEA analysis.

The schematic illustration of the overall research, as shown in Figure 1, involved the evaluation of 374 HCC samples from TCGA-LIHCC to develop a method for indicating PCD expression. Through an initial screening of difference genes (DEGs) and prognostic genes using univariate Cox analysis and a bootstrapping algorithm, a total of 461 prognostic PCDs were identified (Supplementary Table 3). Figure 2A presents the heatmap illustrating the top 20 prognostic PCDs that are up-regulated in HCC. To ascertain the most robust prognostic genes among the candidates, LASSO regression analysis was utilized to determine their risk prediction contributions, as illustrated in Figures 2B, C. Multivariate Cox analysis identified 23 PCD-related genes that significantly influenced PCD scores, as depicted in Figure 2E. Subsequently, patients in the TCGA-LIHC cohort were stratified into two groups on the basis of their PCD scores. Statistical analysis indicated that patients with lower PCD scores performed more favorable clinical outcomes, whereas those with higher PCD scores experienced poorer outcomes (Figure 2D). Furthermore, GSEA revealed a potential distinction between the PCD subgroups, suggesting that tumors characterized by a high PCD phenotype are more proliferative (Figure 2F) and all the enrichment score results could be assessed by Supplementary Table 4. A PCD score was developed to forecast the prognosis of the patients with HCC in this section, and the underlying function of PCD molecules in HCC.

### 3.2 Building the TME score in TCGA-LIHC

**FIGURE 3:**
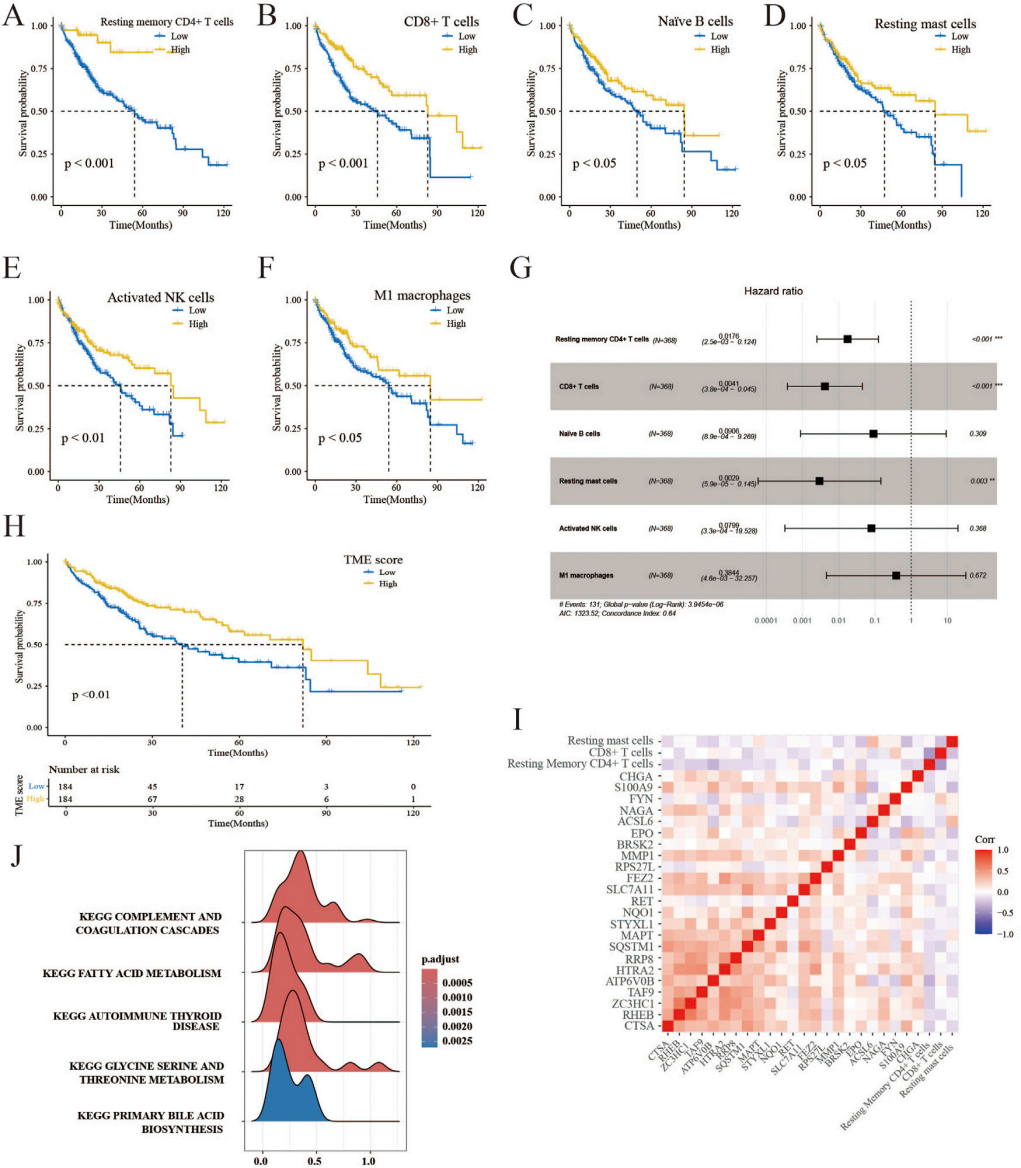
Creation of the TME score and correlation analysis. **(A–F)** K–M survival analysis of HCC patients in low- and high-risk immune cells. **(G)** The forest plot displays a multivariate Cox analysis of immune cells. **(H)** K–M survival analysis of HCC patients in the TME high-and low-score groups. **(I)** Correlation analysis reveals the association between the components of PCD and TME scores. **(J)** GSEA detects phenotype variations in the TME high subgroup.

An immune cell signature was derived from the transcriptomes of 22 immune cells utilizing the CIBERSORT algorithm. Six distinct immune cell types were identified based on their optimal cutoff values, demonstrating their protective roles in OS. These immune cell types consist of resting memory CD4^+^ T cells, CD8^+^ T cells, activated NK cells, naïve B cells, resting mast cells, and M1 macrophages (Figures 3A–F). Figure 3G displays the multivariate Cox analysis of immune cells. It was observed that patients with high TME scores had significantly longer survival compared to those with low TME scores, in contrast to the PCD score, as shown in Figure 3H. Furthermore, Figure 3I presents a correlation analysis demonstrating the association between immune infiltration and PCD expression. The complement system is a group of proteins that promote the removal of microorganisms and damaged cells by antibodies and phagocytes, and it enhances (complements) antibody- and cell-mediated immune mechanisms through a series of cascading reactions, which was enriched in the TME high groups (Figure 3J). The result of the GSEA analysis of TME scores is available in Supplementary Table 5. TME scores and elucidation of the relationship between immune cells and PCD were established in this section.

### 3.3 Establishment of the PCD–TME classifier

**FIGURE 4:**
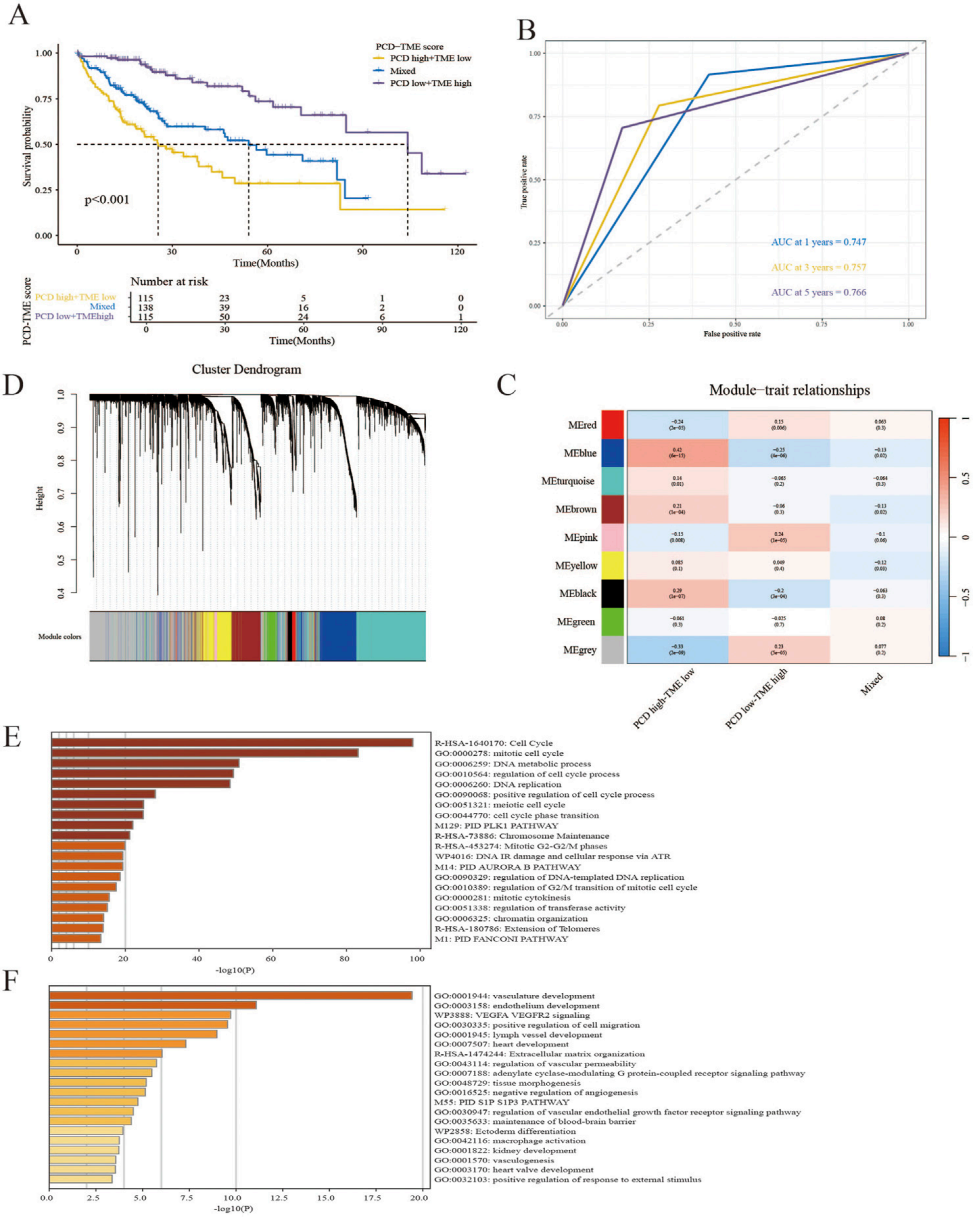
Establishment of the PCD–TME classifier. **(A)** K–M survival analysis for PCD low-TME high, PCD high-TME low and Mixed subgroups. **(B)** The time-dependent ROC curves show the predictive accuracy of the PCD-TME classifier. **(C)** Gene modules from WGCNA reveal distinct clusters in three groups. **(D)** A dendrogram showing gene clusters grouped by similarity into modules. **(E, F)** The top 20 annotations were gathered for the PCD high-TME low group and the PCD low-TME high group.

In light of the aforementioned research, the inquiry arose as to whether a PCD-TME classifier could be developed through the integration of PCD and TME scores. This amalgamation yielded three distinct subgroups: PCD high-TME low, PCD low-TME high and Mixed. A notable disparity in prognosis was observed between the PCD-TME classifier and the TCGA-LIHC cohort, as illustrated in Figure 4A. In contrast to the other two groups, samples in the subgroup characterized by PCD low-TME high exhibit a more favorable prognosis. The time-dependent ROC curves were applied to predict the capability of the PCD-TME classifier. The AUC values were 0.747 for 1 year, 0.757 for 3 years, and 0.766 for 5 years, as depicted in Figure 4B. WGCNA was performed to investigate gene variations among these subgroups in order to elucidate significant differences in survival outcomes. The “pink” and “blue” modules were identified as having the most pronounced variations between PCD low-TME high and PCD high-TME low cohorts, as illustrated in Figures 4C, D. All genes corresponding to the “pink” and “blue” modules were subjected to functional annotation using the Metascape. Enrichment analysis indicated significant enrichment of cell cycle-related pathways in the PCD high-TME low group, whereas tissue development-related pathways were predominantly found in the PCD low-TME high group (Figures 4E, F). We also tested the PCD-TME classifiers by the R package of “fgsea,” which shows a similar result (Supplementary Figure 1). A PCD-TME classifier was developed in this section by integrating PCD and TME scores, and further investigation was conducted to explore the functional distinctions between the two subgroups.

### 3.4 Association between PCD-TME classifier and clinical features

**FIGURE 5:**
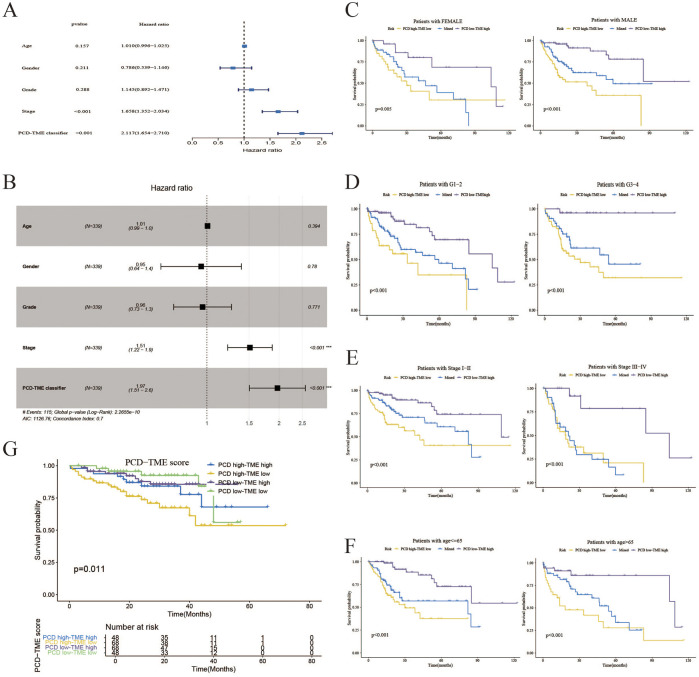
Association between PCD-TME classifier and clinical features. **(A)** A forest plot of univariate analysis demonstrates that the PCD-TME classifier exhibits superior predictive efficacy compared to clinical parameters. **(B)** A forest plot analysis of multivariate data indicates that the PCD-TME classifier serves as an independent prognostic factor for patients with HCC. **(C–F)** The K–M curves of the simplified PCD-TME classifier demonstrate substantial discriminatory ability across various demographic factors, including gender, tumor grade, stage, and age. **(G)** Validation of the PCD-TME classifier in the ICGC-LIRI-JP cohort.

Figures 5A, B illustrate that the PCD-TME classifier performed a statistically significant association with OS in HCC patients, as indicated by a hazard ratio (HR) of 1.97, a 95% confidence interval (CI) of 1.51–2.6, and a *P*-value of less than 0.001, as determined through multivariate Cox analysis. It suggests that PCD-TME classifiers serve as independent prognostic markers for HCC patients. Furthermore, the PCD-TME classifiers demonstrated considerable predictive efficacy across various demographic and clinical factors, including gender, age, stage, and tumor grade, as depicted in Figures 5C–F. Within the ICGC-LIRI-JP dataset, the PCD-TME classifier was confirmed as a significant risk factor in hepatocellular carcinoma (HCC) (Figure 5G). Furthermore, we elucidated the correlation between the PCD-TME classifier and various clinical characters and validated the PCD-TME classifier in an independent cohort within this section.

### 3.5 Differential patterns of TMB and immunotherapy response prediction

**FIGURE 6:**
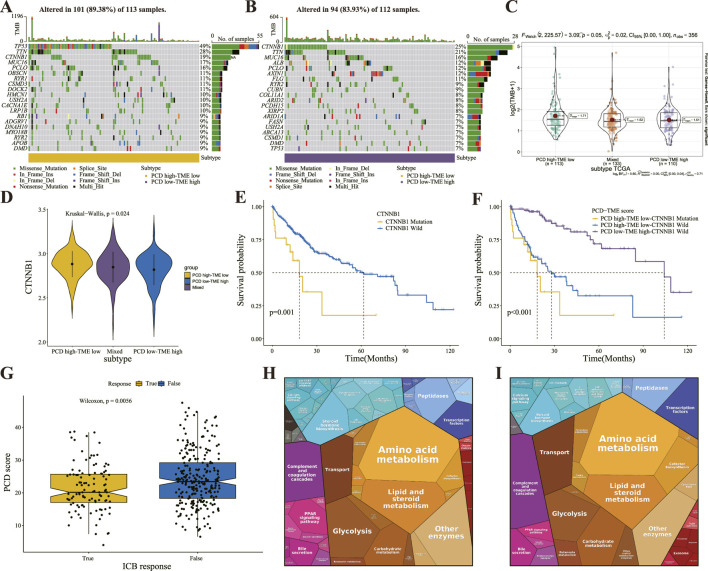
Differential patterns of TMB and immunotherapy response prediction **(A, B)** The OncoPrint illustrates the presence of significant mutation genes in the comparison between PCD high-TME low **(A)** and PCD low-TME high **(B)** groups. **(C)** The difference of TMB in the PCD-TME classifier. **(D)** Comparison of CTNNB1 expression in the PCD-TME classifier. **(E)** K–M survival analysis of HCC patients with or without CTNNB1 gene mutation. **(F)** K–M survival analysis of HCC patients divided by CTNNB1 mutation status and PCD-TME classifier. **(G)** Comparing PCD scores in patients with varying responses to ICB immunotherapy. **(H, I)** Protromaps of functional analysis in the PCD low-TME high **(H)** and ICB immunotherapy **(I)**.

This study investigated somatic alterations within the PCD-TME classifiers, with TCGA-LIHC identifying the 20 most frequent variant mutations (Figures 6A, B). Variations in TMB landscapes were observed between the two subgroups. TP53, TTN, CTNNB1, MUC16, and PCLO were among the top five mutations in the PCD high-TME low group, while CTNNB1, TTN, MUC16, ALB, and PCLO were among the top five in the PCD low-TME high group. Somatic mutations were more prevalent in the PCD low-TME high group compared to those shown in Figures 6A, B. Figure 6C demonstrates a notable distinction in TMB within the PCD-TME classifier. It has been established in previous research that CTNNB1 ranks among the proto-oncogenes exhibiting the highest mutation frequency in hepatocellular carcinomas (22%), with over half of hepatoblastomas presenting CTNNB1 mutations (Cleary et al., 2013; Harding et al., 2019; Nakagawa et al., 2019; Nakagawa and Shibata, 2013; Senni et al., 2019; Zucman-Rossi et al., 2015). The levels of CTNNB1 expression exhibited variability across the PCD-TME classifiers, as illustrated in Figure 6D. Notably, the subgroup characterized by PCD high-TME low exhibited a significantly elevated incidence of CTNNB1 mutation. Previous research has indicated that patients exhibiting low levels of CTNNB1 expression experienced longer survival durations in contrast to those with high CTNNB1 expression levels, which is consistent with our findings (Figure 6E). A comprehensive examination was conducted utilizing Kaplan-Meier curves in conjunction with CTNNB1 and PCD-TME subgroups. Notably, the classifier demonstrated the ability to discern patients with more favorable prognoses within the subset of individuals harboring CTNNB1 mutations (Figure 6F). These results suggest that the PCD-TME classifier exhibits heightened sensitivity in patient stratification and can effectively pinpoint improved prognostic outcomes in individuals with CTNNB1 mutations. Our hypothesis posited that the PCD-TME classifier could effectively forecast clinical responses in immunotherapy patients due to differences in immune statuses and tumor mutational burdens. To investigate this hypothesis, the TIDE algorithm was employed to predict responses to immunotherapy. Among patients with HCC who exhibited positive responses to immune checkpoint blockade (ICB) therapy, PCD scores were found to be significantly lower (Figure 6G). Additionally, the Proteomap tool was utilized to visually elucidate the potential mechanism through which the PCD-TME classifier predicts immunotherapy responses. As depicted in Figures 6H, I, Proteomap patterns exhibited striking similarities between individuals with PCD low-TME high levels, as well as those who responded favorably to immunotherapy. Furthermore, a notable resemblance was noted between the PCD high-TME low subgroup and the immunotherapy nonresponder (Supplementary Figure 2). In conclusion, these findings imply that a pretreatment PCD-TME signature could serve as a potential indicator of the patient’s tumor immune microenvironment, thereby aiding in the prediction of their therapeutic response.

### 3.6 Single-cell verification of PCD-TME scores

**FIGURE 7:**
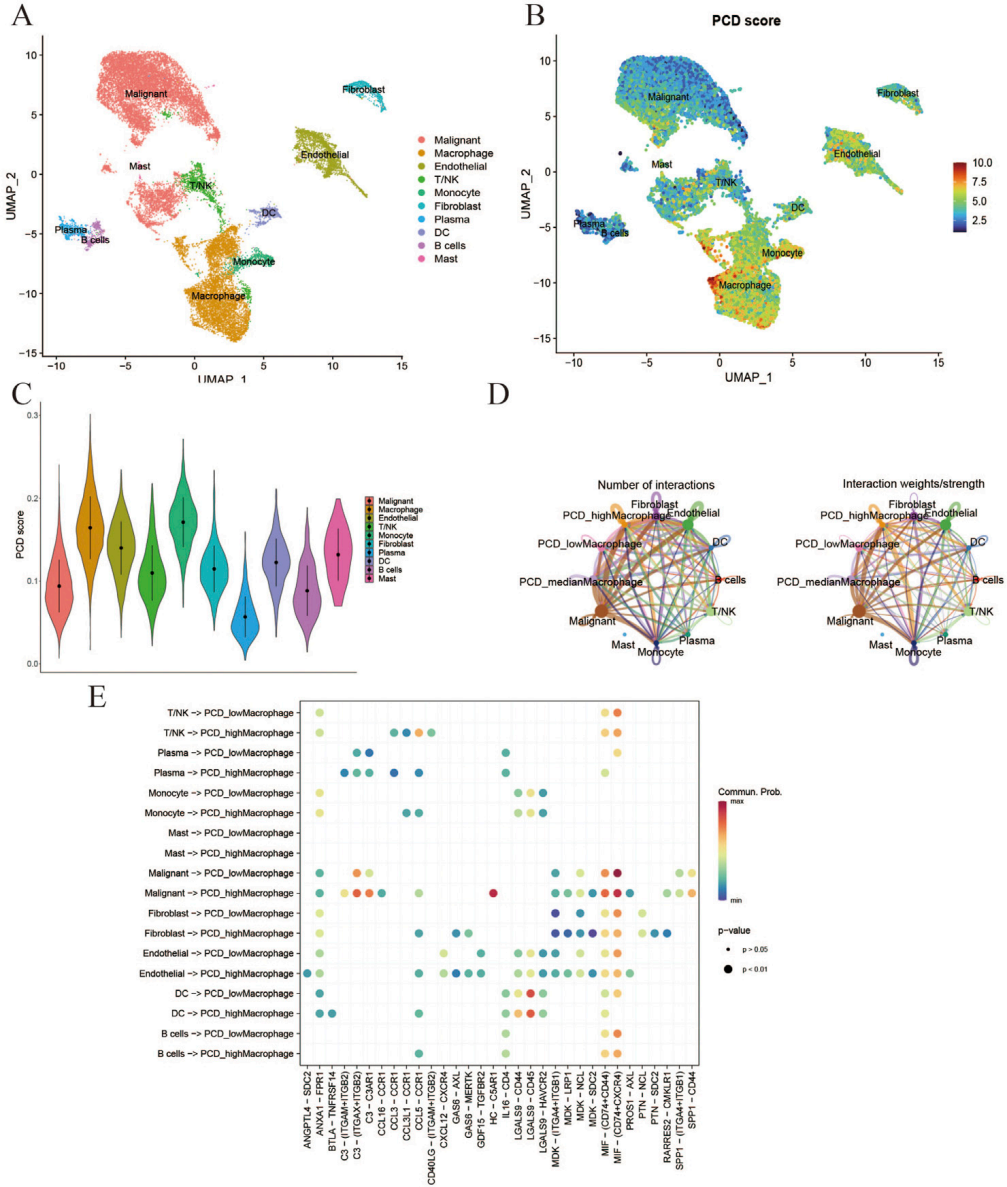
Verification of PCD-TME scores at single-cell level. **(A)** UMAP plot for identification of 10 types of cells in GSE242889. **(B, C)** Visualization of PCD scores at the single-cell level through the utilization of feature plots and violin plots. **(D)** The quantification of interactions for the analysis of intercellular communication. **(E)** The activation of the cell signaling pathway is compromised by the division of macrophages as indicated by the PCD score.

Additionally, the validity of the PCD score was confirmed through single-cell transcriptomic analysis. Following quality control, a total of 19 cell clusters (Supplementary Figure 3) were identified from five samples. Subsequently, ten distinct cell types (Malignant, Macrophage, Endothelial, T/NK, Monocyte, Fibroblast, Plasma, DC, B cells, and Mast) were distinguished using marker genes (Figure 7A). The “AddModuleScore” function was utilized to compute PCD scores for individual cells, indicating that immune cells (Macrophages, Monocytes, and Mast Cells) displayed elevated PCD scores (Figures 7B, C). The examination of intercellular communication among immune cells was conducted to assess the potential impact of PCD status on their functionality. Macrophages were specifically chosen for further analysis based on the results depicted in Figure 7C. Figure 7D depicts the extent of interactions and the intensity of intercellular communication. Figure 7E shows that immune cells (macrophages) with a high expression of PCD are more likely to activate intercellular signaling with malignant cells. These findings highlight the pivotal role of PCD in modulating immune cell interactions, particularly macrophage-driven communication with malignant cells, suggesting PCD status may influence tumor-immune dynamics.

### 3.7 Clinical validation of PCD-related gene

**FIGURE 8:**
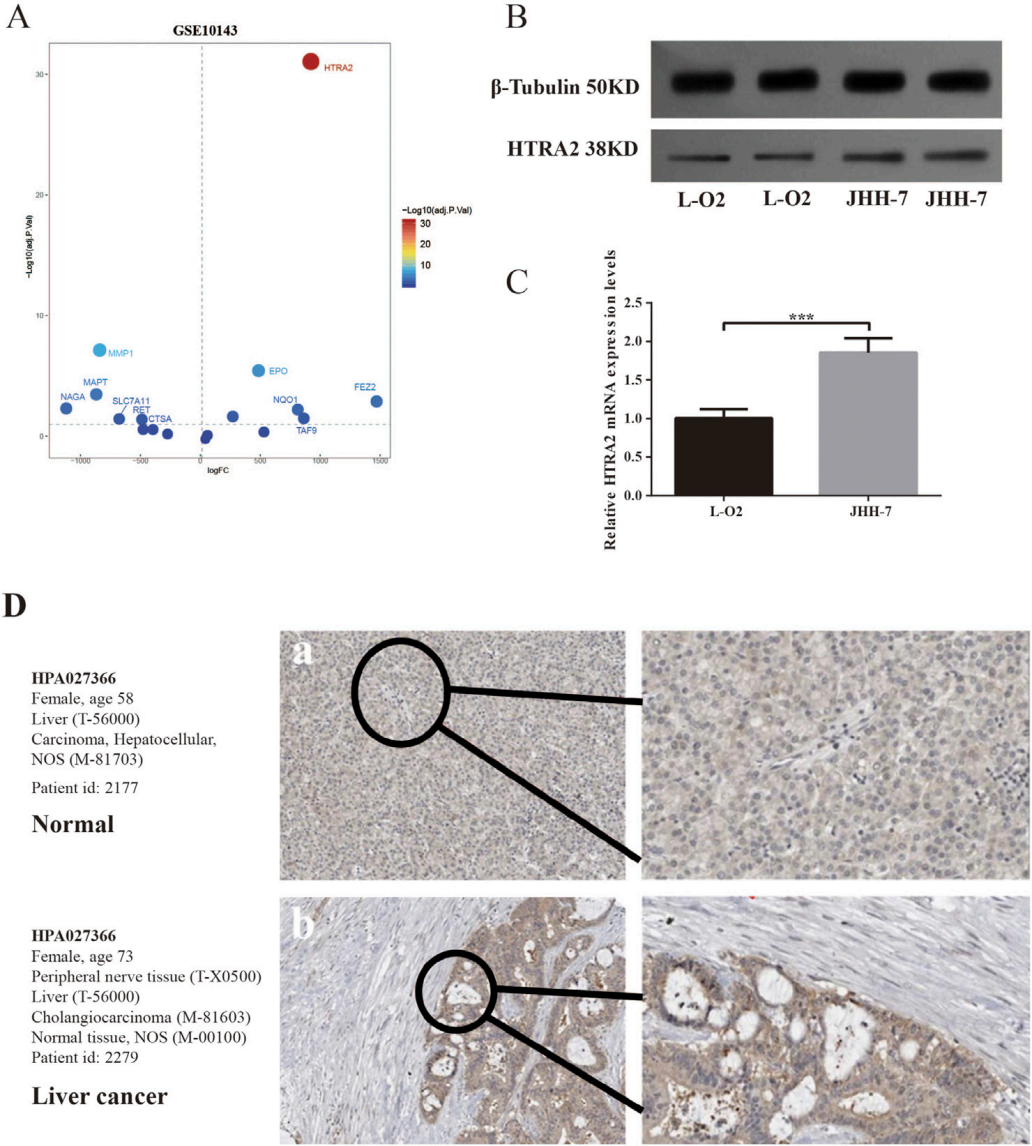
Application of GEO filtration to identify DEGs associated with PCD and subsequent validation. **(A)** The volcano plot depicting the DEGs associated with PCD intersected by the TCGA-LIHC and GSE10143. **(B)** Western blotting was used to measure the expression of HTRA in JHH-7 HCC cells and L-O2 normal liver cells. **(C)** qRT-PCR of HTRA2 gene expression in JHH-7 HCC cells and L-O2 normal liver cells. **(D)** The protein expression levels of HTRA2 in HCC and normal tissue were analyzed using data from the HPA database.

To further investigate potential PCD-related genes involved in regulating HCC development, the GEO dataset GSE10143 was utilized for cross-DEG analysis. Our findings reveal a significant upregulation of HTRA2 in HCC tissues compared to adjacent paracancerous, as illustrated in Figure 8A. Consistent results were achieved via WB, thereby validating the observed differential expression of HTRA2 within the overlapping DEGs (Figure 8B). The results of qRT-PCRT performed on L-O2 normal liver cells and JHH-7 HCC cells also show the similar results suggested by the crossed DEGs (Figure 8C). The HPA database was used to examine HTRA2 protein expression at the protein level. In the HPA database, there is a relatively high expression of HTRA2 in HCC when compared to normal (Figure 8D).

## 4 Discussion

Recent studies on PCDs and their interaction with the TME have contributed to a deeper comprehension of their pivotal roles in cancer prognosis and the development of treatment approaches. However, a limited number of studies incorporate multi-omics data to predict response rates to immunotherapy and OS in relation to PCDs and the TME. Our comprehensive study extensively investigated the crosstalk between PCDs and the TME by integrating diverse HCC datasets. The PCD–TME classifier, developed as a result of this study, has demonstrated significant efficacy in predicting OS and responses in HCC patients.

The subgroup characterized by PCD low-TME high demonstrated the most favorable prognosis. Additionally, the prognostic efficacy of the classifier was validated in an external independent dataset, suggesting its potential applicability to HCC patients. Shared characteristics may exist in the immunotherapy response of PCDs and TMEs. In our study, 23 PCD-related genes were included in our risk signature, such as Ras Homolog Enriched in Brain (RHEB), Sequestosome 1 (SQSTM1), Solute Carrier Family 7 Member 11 (SLC7A11), Microtubule-Associated Protein Tau (MAPT), Matrix Metallopeptidase 1 (MMP1), and HtrA Serine Peptidase 2 (HTRA2). These genes have been extensively investigated, with RHEB identified as a direct activator of mTORC1 (mTOR complex 1) (Yang et al., 2017). The mTOR pathway is a crucial regulatory pathway found in various cancer types, affecting proliferation and survival (Soave et al., 2016). Elevated levels of RHEB or abnormal activation of the mTORC1 signal pathway have been linked to increased tumor proliferation (Maiuri et al., 2009). Furthermore, mTORC1 activation suppresses autophagy, a cellular process involved in self-degradation and recycling. In tumor cells, autophagy inhibition hinders the cells’ ability to protect themselves through this pathway in response to nutrient starvation, ultimately facilitating tumor cell survival and growth (Chen et al., 2012; Maiuri et al., 2009). RHEB has been found to impact the tumor microenvironment by modulating the mTORC1 signaling pathway. Activation of mTORC1 has been shown to stimulate angiogenesis, leading to increased nutrient and oxygen supply to tumors (Advani, 2010). Moreover, mTORC1 regulates immune cell function and influences tumor immune evasion (Ekshyyan et al., 2013). Currently, the development of therapeutics targeting RHEB or the mTORC1 signaling pathway is a significant focus in anticancer research. For instance, compounds like rapamycin and its derivatives have demonstrated efficacy in inhibiting tumor growth by targeting mTORC1 activity, making them promising candidates for cancer treatment (Xu et al., 2020). SQSTM1, also referred to as Sequestosome 1 or p62, serves as a crucial adapter protein in the process of autophagy by binding to ubiquitinated proteins and directing them to the autophagosome for degradation (Deng et al., 2020). Moreover, SQSTM1 can modulate the cellular response to oxidative stress and bolster the antioxidant capabilities of the cell through its interaction with NRF2 (Shi et al., 2022). Additionally, SQSTM1 is implicated in the regulation of various signaling pathways, such as NF-KB and mTOR, which have profound effects on cell proliferation, survival, and inflammatory reactions (Liu et al., 2022; Sultana et al., 2021). It also regulates the mitochondria-mediated apoptosis pathway, inhibiting apoptosis and promoting tumor cell survival and drug resistance (Gao et al., 2017). In the TME, SQSTM1 regulates the function of tumor-associated macrophages, affects immune escape and inflammatory responses in tumors, and promotes malignant invasion and metastasis by regulating extracellular matrix (ECM) degradation (Qi et al., 2021). The significance of MAPT in tumor progression is primarily demonstrated through its influence on cytoskeletal regulation (Papin and Paganetti, 2020). MAPT modulates cell morphology, migration, and division by stabilizing microtubule structure (Sferra et al., 2020). Dysregulation or mutation of MAPT can result in cytoskeletal reorganization, thereby promoting enhanced migration and invasiveness of tumor cells (Wesley et al., 2021). Research has found a correlation between MAPT expression levels and tumor aggressiveness in specific cancers, such as breast and prostate cancer (Ma et al., 2020). NQO1, functioning as an antioxidant enzyme, is a cellular defense against oxidative stress and mitigation of oxidative damage through the reduction of oxidized quinones to non-toxic hydroquinones (Tejchman et al., 2021). Elevated levels of NQO1 in tumor cells bolster the antioxidant capabilities of cells, thereby fostering tumor cell survival in adverse conditions (Ross and Siegel, 2021). Furthermore, NQO1 is implicated in cellular metabolism and signal transduction pathways, impacting tumor cell proliferation and PCD. SLC7A11 primarily influences tumor cell viability by modulating the cellular response to oxidative stress (Yan et al., 2023). SLC7A11, as the light chain subunit of system Xc-, facilitates the transport of extracellular cysteine into the cell and glutamate out of the cell (Dahlmanns et al., 2023). Cysteine serves as a precursor for the synthesis of glutathione (GSH), an essential intracellular antioxidant. Through the promotion of GSH synthesis, SLC7A11 aids tumor cells in resisting oxidative stress and cytotoxicity induced by chemotherapeutic drugs, thereby facilitating tumor cell proliferation and survival (Zhao et al., 2022). Furthermore, elevated expression of SLC7A11 has been linked to increased aggressiveness and drug resistance in various types of cancers (Sun et al., 2022). MMP1 facilitates tumor cell invasion and metastasis predominantly by ECM degradation, particularly collagen and other ECM components, thereby enabling tumor cells to breach the basement membrane and infiltrate adjacent tissues (Winkler et al., 2020). Moreover, MMP1 can stimulate tumor cell proliferation and angiogenesis by liberating growth factors and cytokines from the ECM, thereby enhancing the availability of oxygen and nutrients to malignant (Al-Ostoot et al., 2021). HTRA2, a member of the hyperthermia-requiring family of serine proteases localized in mitochondria (Vande Walle et al., 2008), was initially believed to be a heat-shock-induced serine protease in *Escherichia coli*. However, it has since been recognized as a pro-apoptotic protein of the mitochondrion, playing a role in the regulation of mitochondrial homeostasis (Xu et al., 2018). Many researches have indicated the involvement of HTRA2 in the pathogenesis of various cancers, like ovarian, breast, colorectal, and prostate cancers (Wang and Nie, 2021). Recent research has demonstrated the possibilities of plasma HTRA2 as a clinical diagnostic biomarker for gastric cancer (Rosochowicz et al., 2024). The role of HTRA2 in inducing apoptosis in hepatocellular carcinoma is contingent upon its expression levels (Feng et al., 2023). Furthermore, HTRA2 has been implicated in the suppression of hepatocellular carcinoma cell proliferation through its interaction with astrocystin (Ding et al., 2017). The upregulation of HTRA2 within the tumor microenvironment of hepatocellular carcinoma warrants further exploration, as supported by analysis of the GEO dataset and examination of clinical tissue specimens in our investigation.

In the alternate segment of the classifier, the TME score is deemed significant in combating cancer. This particular model has recognized CD4^+^ T cells, CD8^+^ T cells, resting mast cells, naive B cells, activated NK cells, and M1 macrophages as protectors of the HCC TME. The anti-tumor efficacy of memory CD4 cells, CD8 cells, naive B cells, activated NK cells, and M1 macrophage cells has been validated across various cancer types. Nevertheless, limited research has been performed on the involvement of resting mast cells in the HCC tumor microenvironment. A large-scale study involving 245 patients with HCC found higher levels of mast cell infiltration in HCC samples and better OS after surgery resection (Lin et al., 2013). Researchers also found PT CD117+ mast cells were significantly related to longer OS in patients with colorectal liver metastases (Giuşcă et al., 2015). Rohr-Udilova et al. (2018) reported a greater density of mast cells in the adjacent tissue of HCC; however, only the density of intra-tumoral mast cells was found to be associated with a reduced risk of recurrence. The study shows that mast cells are largely inactive in HCC (Giuşcă et al., 2015). Since activation of mast cells by IgE is thought to prevent the development of cancer, deactivation of mast cells could lead to immune escape, thereby promoting tumor growth (Rohr-Udilova et al., 2018). Mast cells serve as regulators of immune effector cells, making them a promising target for immunotherapy. Their abundance and immobility in the liver and tumors, along with their relative radioresistance and resistance to chemotherapeutic agents compared to other rapidly dividing immune cells, make mast cells an attractive target for therapeutic intervention.

Utilizing the PCD–TME classification system, a novel prognostic signature was developed. The best prognosis and response to immune checkpoint blockade treatment were observed within the cohort of HCC patients categorized as PCD low-TME high. Time-dependent ROC curves were used to validate the sensitivity and specificity of this risk signature, highlighting PCD-TME as an independent prognostic factor. Integrating the PCD-TME classifier can potentially improve the precision of molecular subtyping and treatment approaches in clinical practice. Furthermore, the application of bulk-seq following surgical intervention is feasible. Gene expression data could be utilized to derive PCD and TME scores, aiding in the classification of patients into distinct PCD-TME subgroups for the prediction of overall survival and response to immunotherapy. However, our study is constrained by limitations, including the reliance on retrospective datasets from public databases introduces inherent biases, such as variability in clinicopathologic characteristics, sample handling, and sequencing technologies across different cohorts. These variations may affect the generalizability of our findings and the robustness of the PCD-TME classifier. Additionally, selection bias could arise due to the inclusion criteria for patients in these databases, which may not represent the full diversity of HCC cases in clinical settings. This could skew the prognostic significance of the PCD-TME signature and limit its applicability across broader patient populations. To overcome these limitations and strengthen the clinical relevance of our findings, large-scale, randomized, multicenter prospective trials are necessary. These trials would help validate the prognostic accuracy of the PCD-TME classifier and confirm its utility in predicting patient outcomes and response to immunotherapy. Additionally, experimental validation of key prognosis-related genes identified in our study should be conducted in preclinical models, such as *in vitro* cell lines and *in vivo* animal studies. This approach would provide further mechanistic insights into how PCD and TME interact to drive tumor progression and response to treatment. Overall, while our study provides a promising framework for integrating PCD-TME into clinical practice, further research is essential to validate its prognostic potential and refine its application in precision oncology.

Cancer immunotherapy has emerged as a significant treatment modality for various cancers, leading to notable complete and enduring responses. However, the efficacy of this therapy is constrained by the restricted immune activation against tumor-specific antigens, resulting in a limited response rate among patients with specific cancer types. Therefore, the identification of alternative therapeutic targets is imperative in advancing cancer treatment strategies. This study demonstrates a significant association between the PCD-TME classifier and the prognosis of the patients with HCC. Consequently, our research offers a novel therapeutic strategy for HCC treatment, with potential implications for the advancement of cancer therapies.

## 5 Conclusion

Based on our study findings, the integration of tumor microenvironment landscape signatures and programmed cell death markers shows promise in enhancing prognostic accuracy and predicting immunotherapy response in individuals diagnosed with HCC. This methodology could prove to be a valuable asset for prognostic assessment and risk stratification of HCC patients within clinical settings.

## Data Availability

The original contributions presented in the study are included in the article/Supplementary Material, further inquiries can be directed to the corresponding author.
