# Supplementary material for: The combined signatures of programmed cell death and immune landscape provide a prognostic and therapeutic biomarker in the hepatocellular carcinoma
Source: Front Chem. 2024 Nov 12;12:1484310. doi: 10.3389/fchem.2024.1484310 (PMC11591233; doi:10.3389/fchem.2024.1484310)
Supplement: Supplementary file 2 [file DataSheet1.docx]

Supplementary Material

**
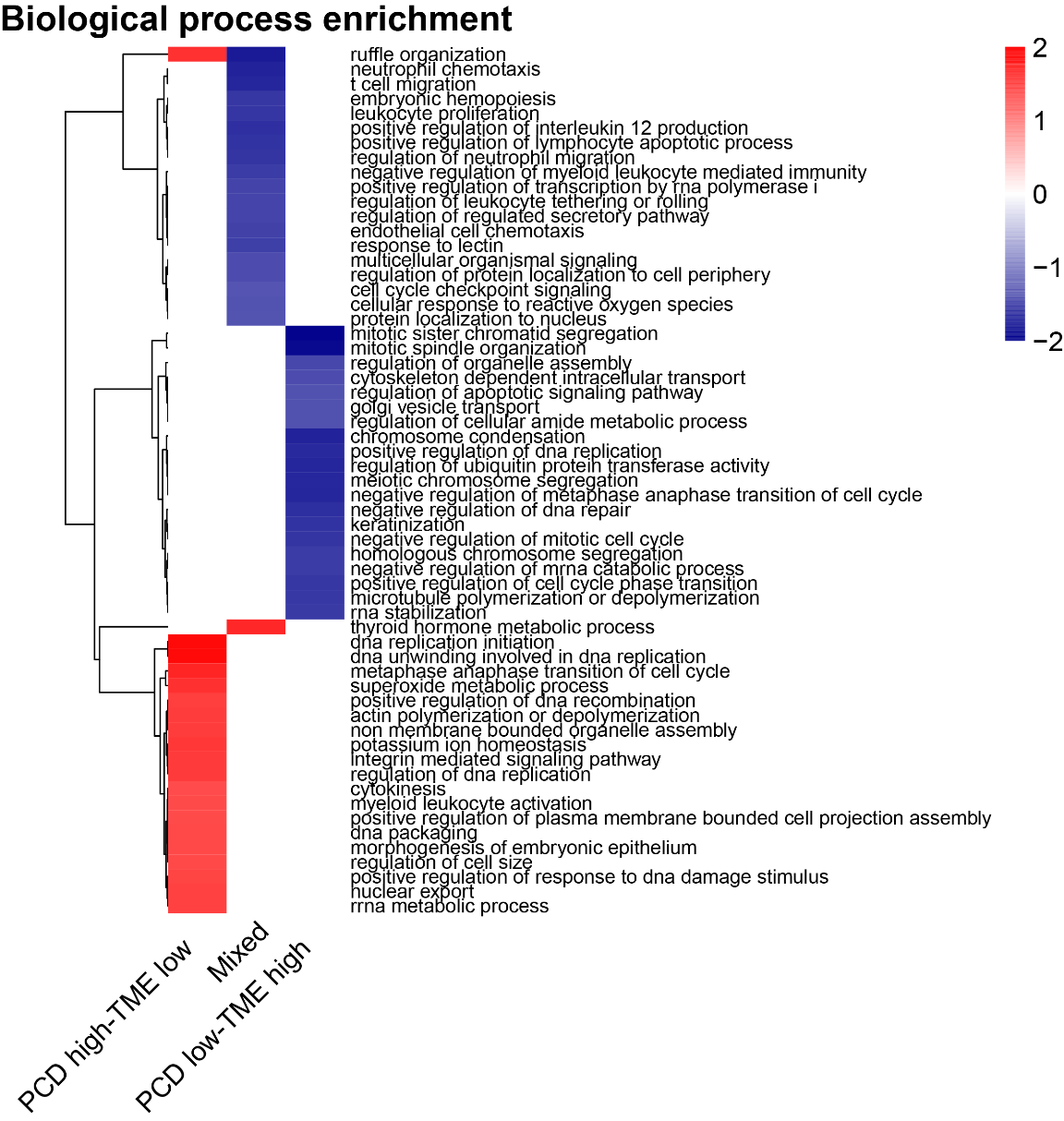
**

**Supplementary Figure. 1** The fgsea analysis of the PCD-TME classifier.

**
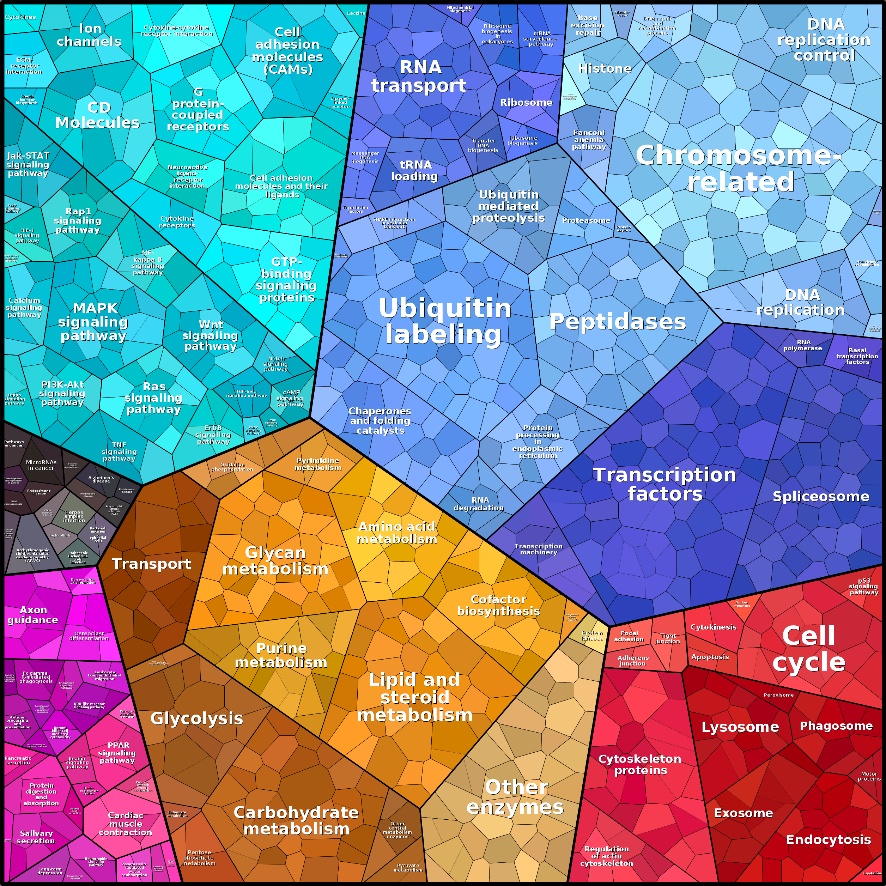

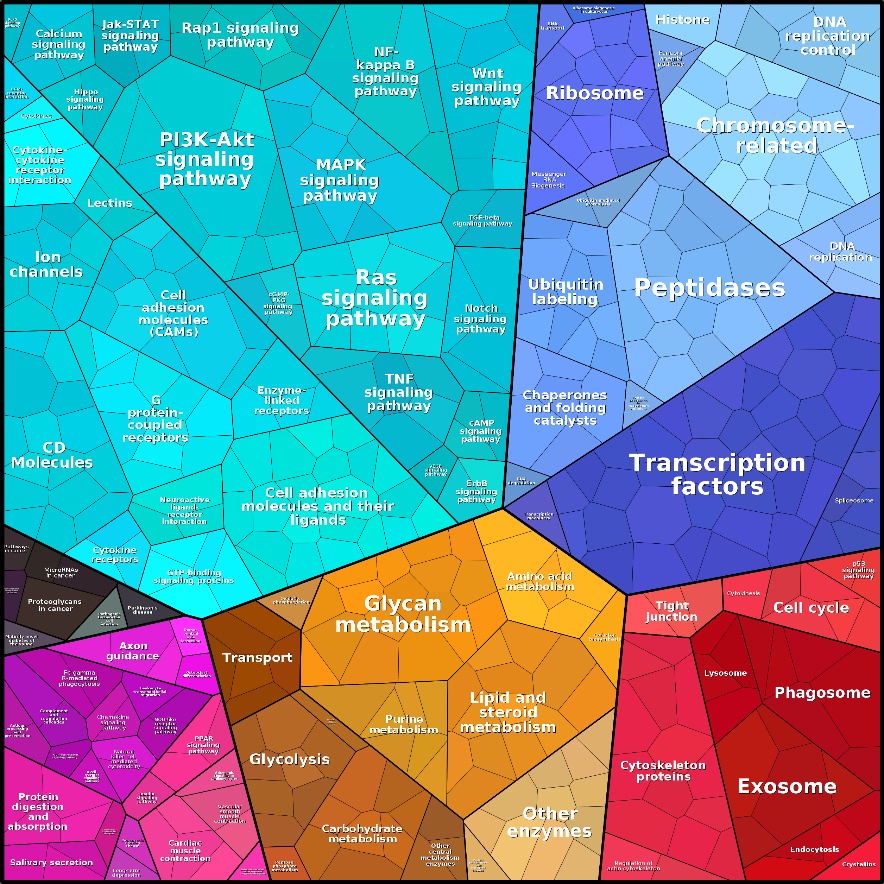
**

**Supplementary Figure 2.** Protromaps of functional analysis in the PCD high-TME low (left) and ICB immunotherapy nonresponder (right).

**
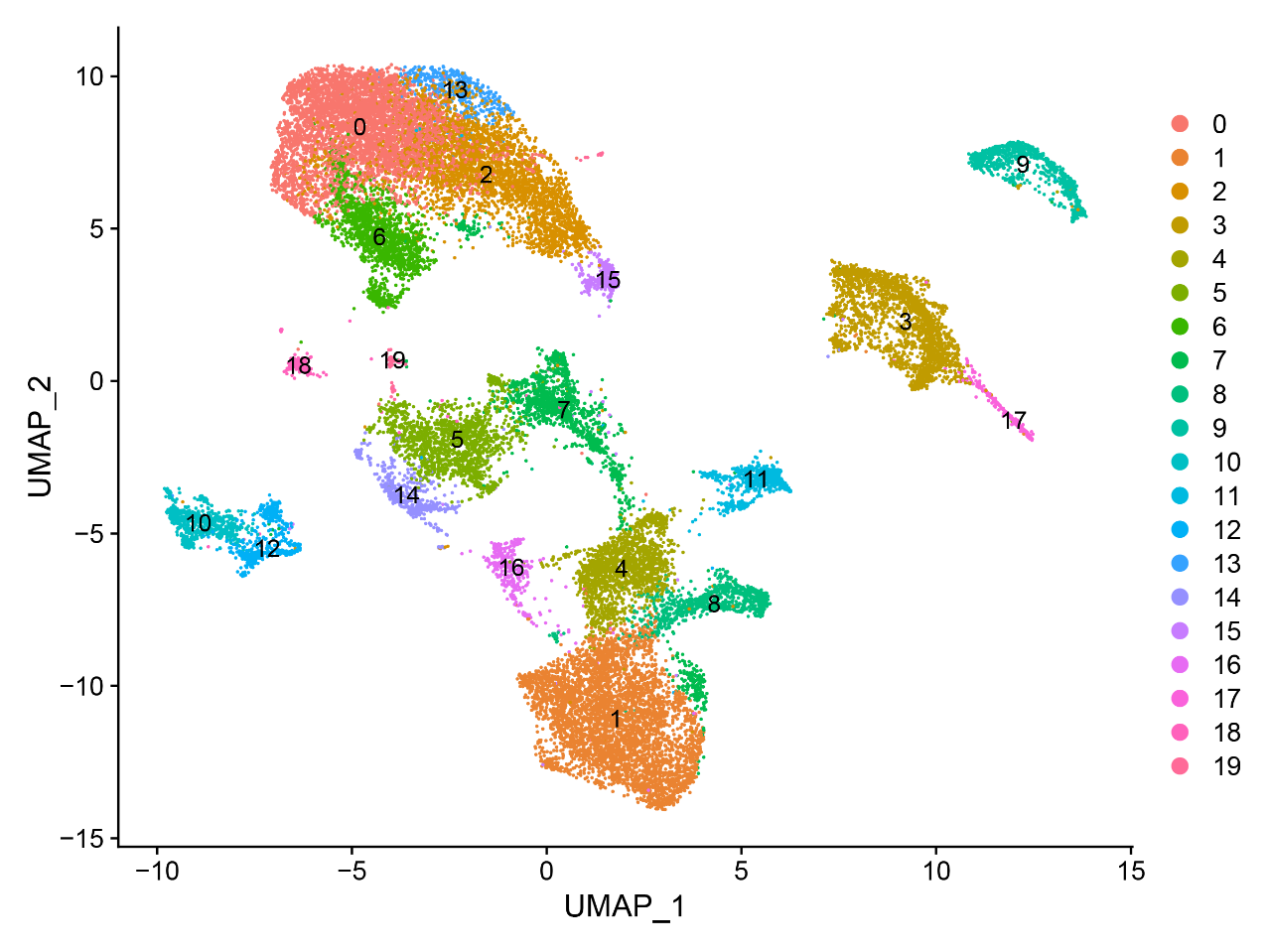
**

**Supplementary Figure 3.** The umap displays 19 clusters.

**
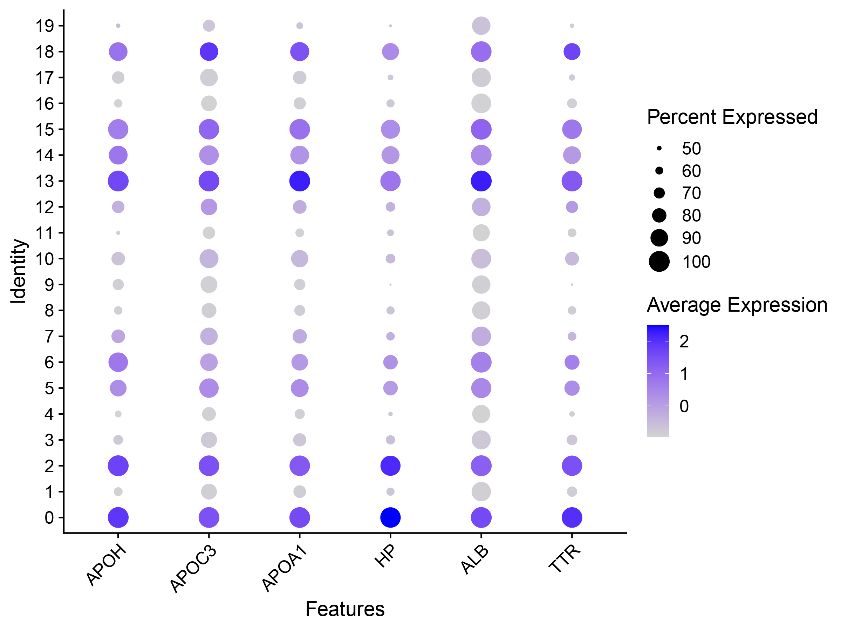
**

**(A) Malignant**

**
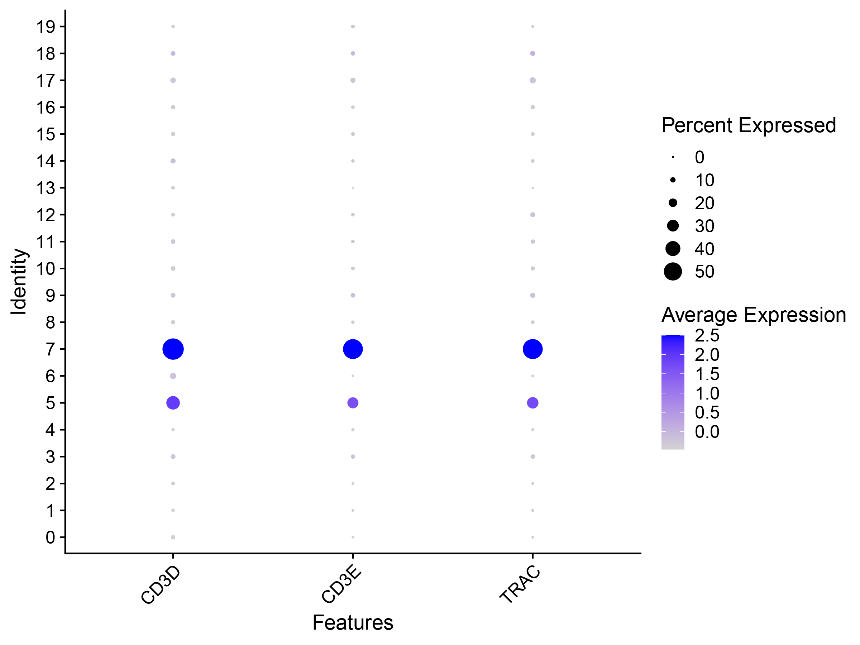
**

**(B) T_cell**

**Supplementary Figure 4.** *Continued.*

**
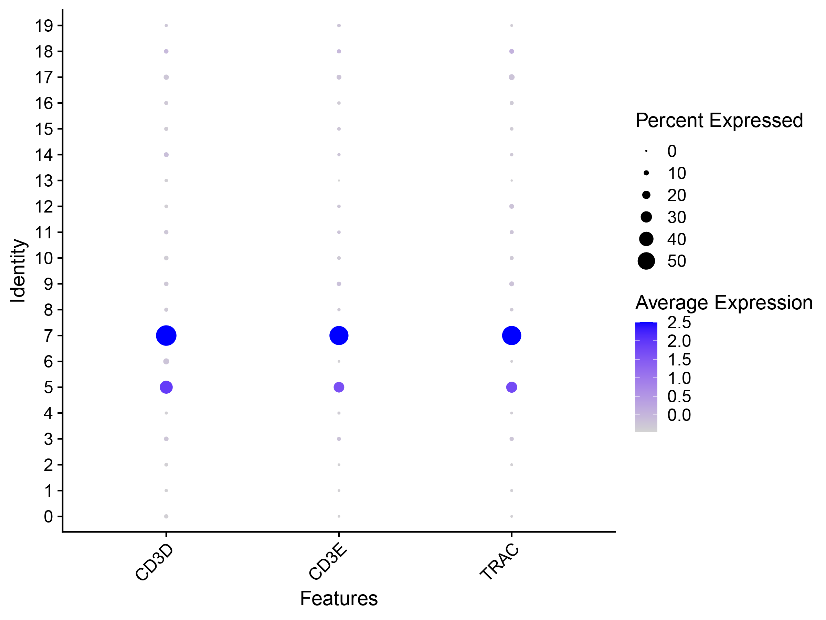
**

**(C) B_cell**

**
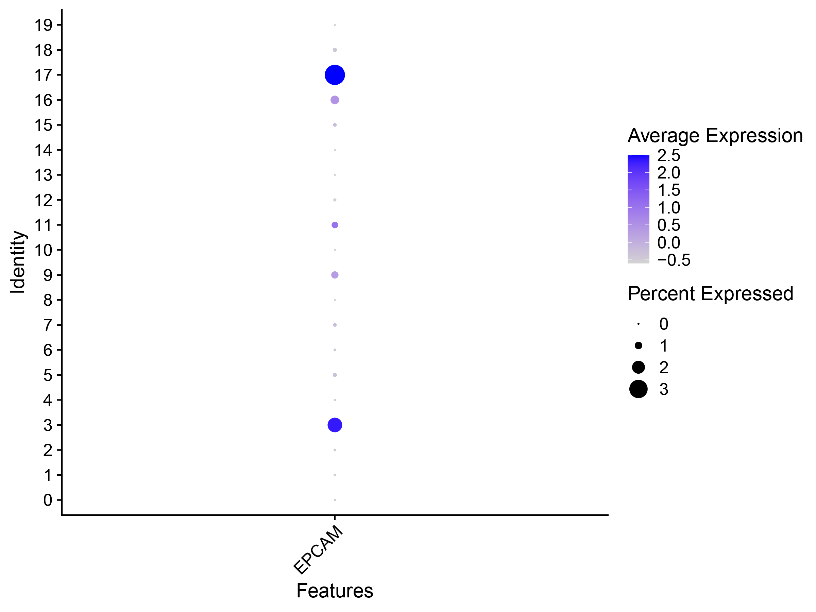
**

**(D) Epithelial_cell**

**Supplementary Figure 4.** *Continued.*

**
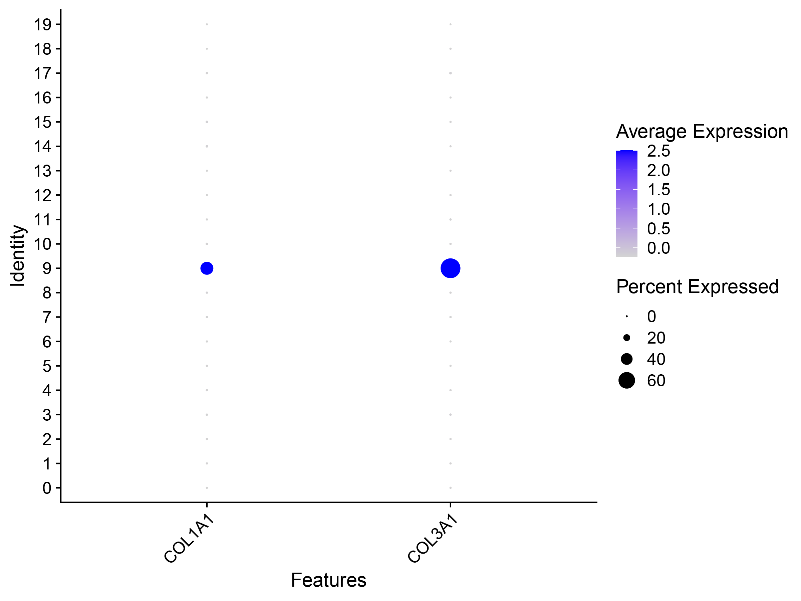
**

**(E) Fibroblast**

**
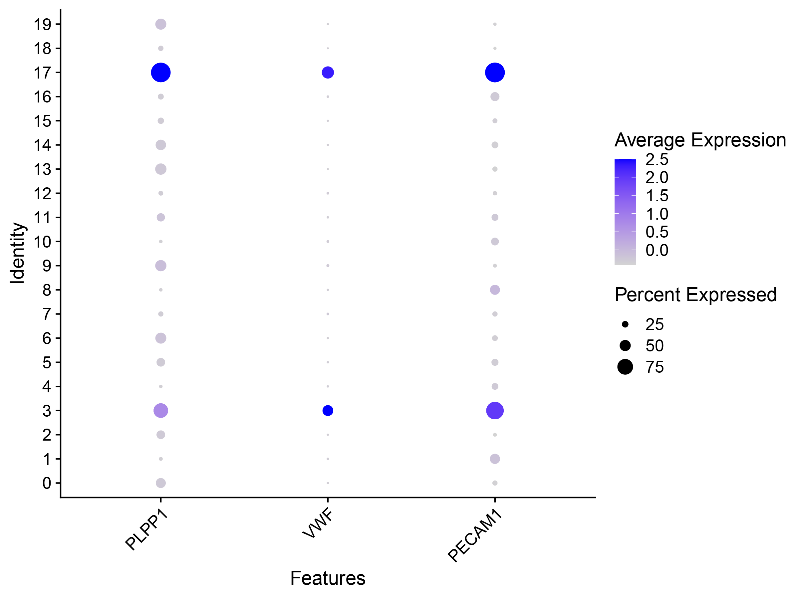
**

**(F) Endothelial**

**Supplementary Figure 4.** *Continued.*

**
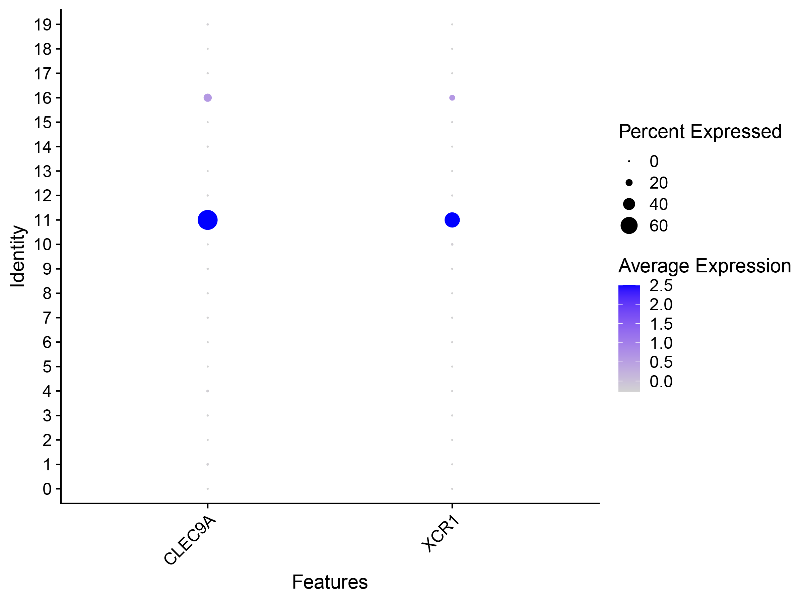
**

**(G) DC**

**
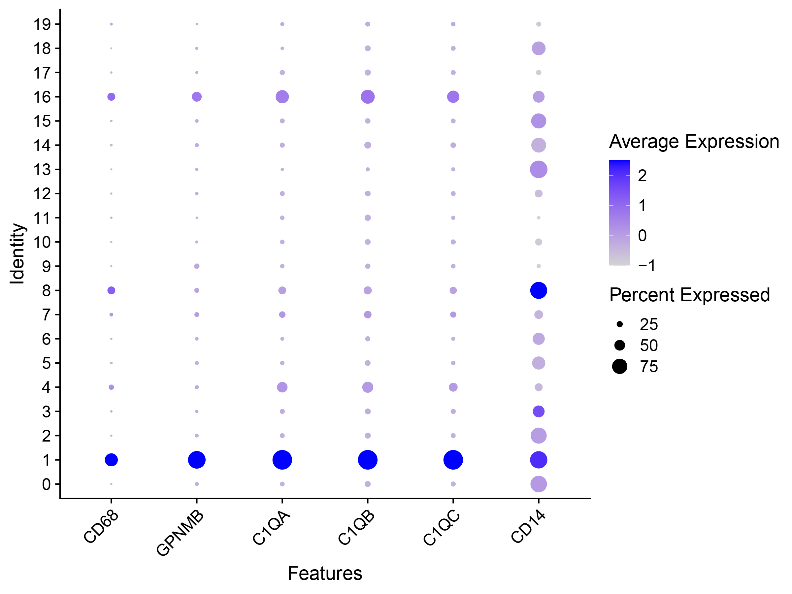
**

**(H) Macrophage**

**Supplementary Figure 4.** *Continued.*

**
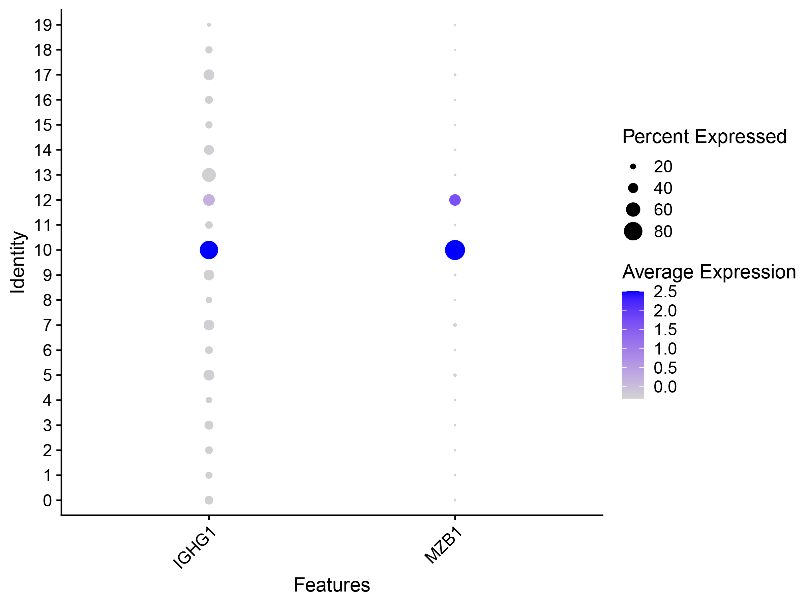
**

**(I) Plasma**

**
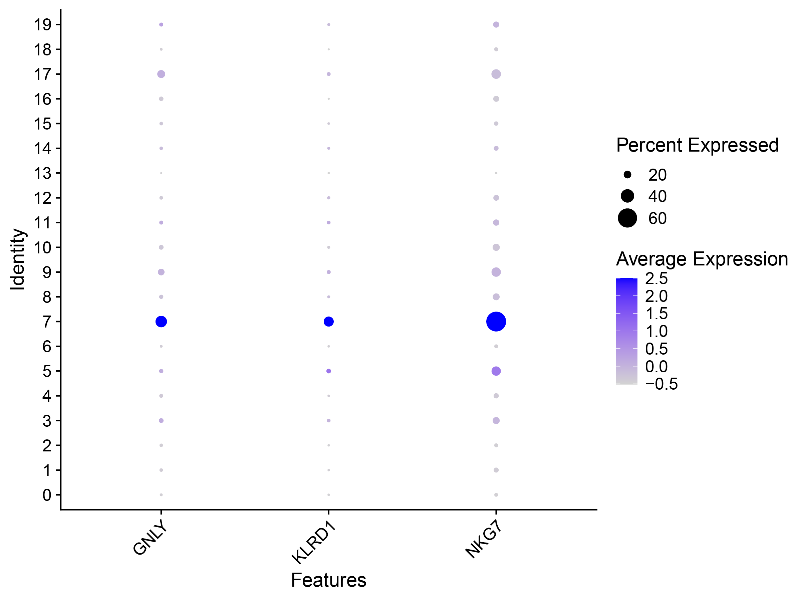
**

**(J) NK**

**Supplementary Figure 4.** *Continued.*

**
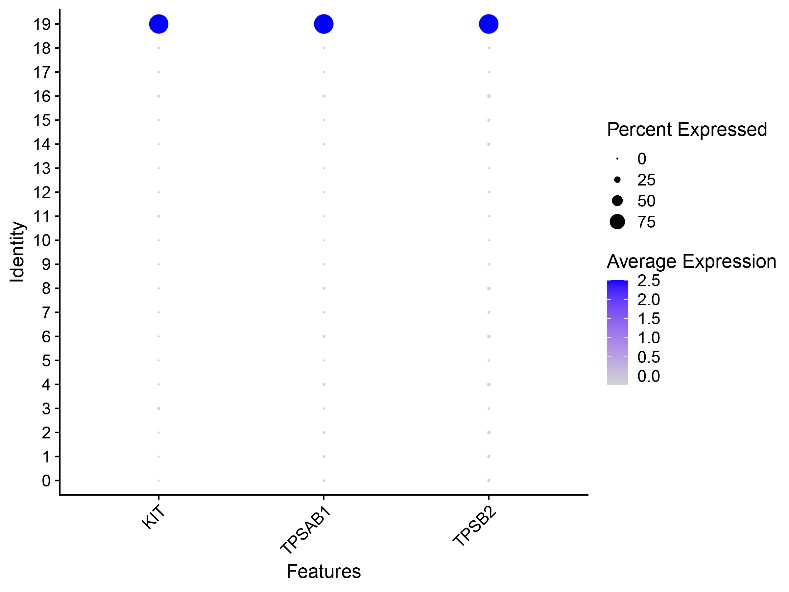
**

**(K) Mast**

**Supplementary Figure 4.** The DotPlot and FeaturePlot of each cell type.


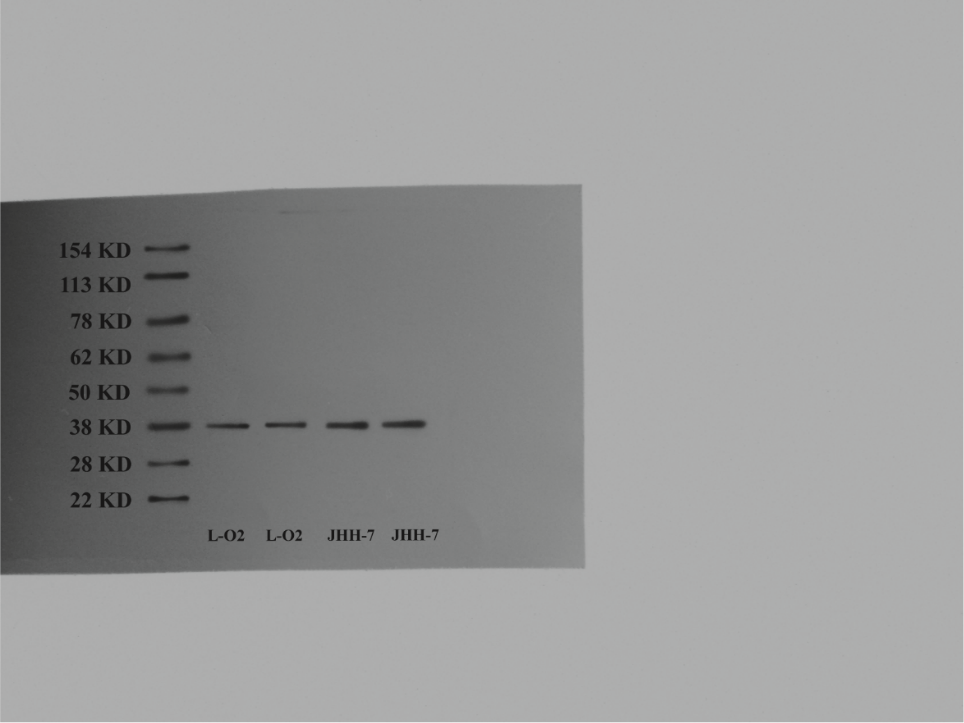


**Supplementary Figure 5.** The original WB picture of HTRA2.


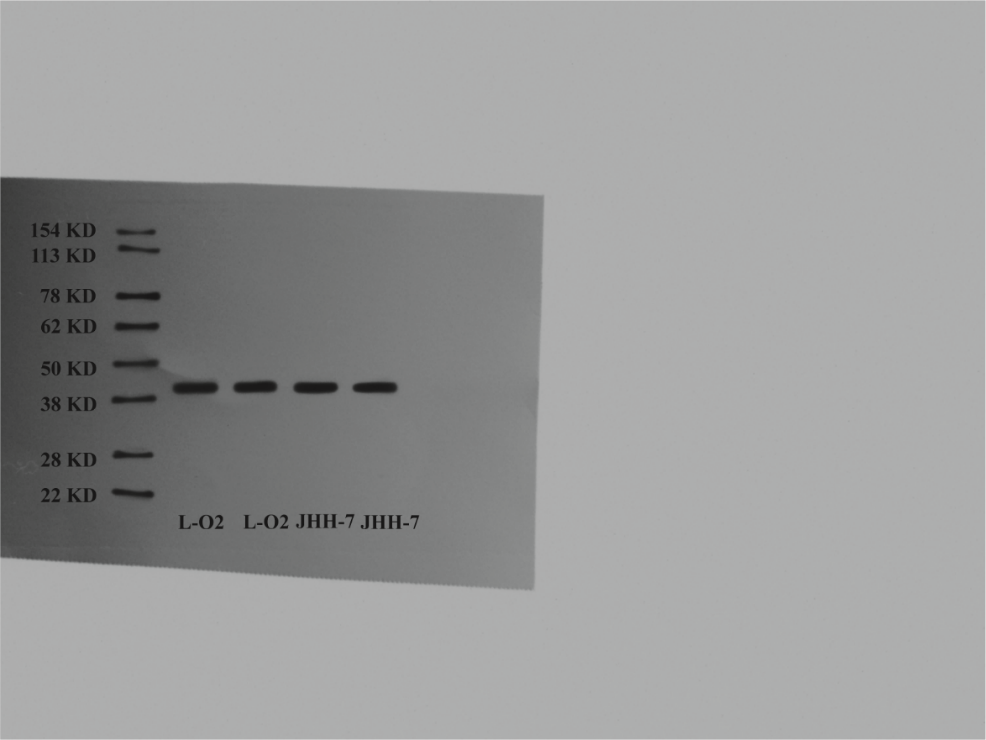


**Supplementary Figure 6.** The original WB picture of β-actin.
